# Seroprevalence and risk factors for HIV, HCV, HBV and syphilis among blood donors in Mali

**DOI:** 10.1186/s12879-019-4699-3

**Published:** 2019-12-19

**Authors:** Aude Jary, Sidi Dienta, Valentin Leducq, Quentin Le Hingrat, Mahamadou Cisse, Amadou B. Diarra, Djeneba B. Fofana, Alhassane Ba, Mounirou Baby, Chad J. Achenbach, Robert Murphy, Vincent Calvez, Anne-Geneviève Marcelin, Almoustapha I. Maiga

**Affiliations:** 1 Department of Virology, Sorbonne Université, INSERM, Institut Pierre Louis d’Epidémiologie et de Santé Publique (iPLESP), AP-HP, Pitié Salpêtrière Hospital, Paris, France; 2 Département de Biologie Médicale, CHU Gabriel Toure, Bamako, Mali; 3 Department of Virology, IAME, UMR 1137, INSERM, Université Paris Diderot, Sorbonne Paris Cité, Bichat Hospital, APHP, Paris, France; 4 Centre National de Transfusion Sanguine, Bamako, Mali; 5 Department of Virology, Sorbonne Université, INSERM, Institut Pierre Louis d’Epidémiologie et de Santé Publique (iPLESP), AP-HP, Saint-Antoine Hospital, Paris, France; 6 Northwestern University, Institute for Global Health, Chicago, IL USA; 7 Université des Sciences Techniques et des Technologies de Bamako, USTTB, Centre de Recherche et de Formation sur le VIH et la Tuberculose SEREFO, Bamako, Mali

**Keywords:** HIV-prevalence, HBV-prevalence, HCV-prevalence, Blood donors, Mali

## Abstract

**Background:**

HIV, HBV and HCV remain a global public health concern especially in Africa. Prevalence of these infections is changing and identification of risk factors associated with each infection in Mali is needed to improve medical care.

**Methods:**

We conducted a cross-sectional study of all individuals donating blood (*n* = 8207) in 2018 to the blood bank at university hospital in Bamako, Mali, to assess prevalence and risks factors associated with HIV, HBV, HCV and syphilis infections.

**Results:**

HIV-seroprevalence was 2.16% and significantly increased with age, being married and decreasing education level. In multivariate analysis, after adjustements with age, marital status and geographical setting, only education level was associated with HIV-infection (OR, 1.54 [95% CI, 1.15–2.07], *p = 0.016*). HBsAg prevalence was 14.78% and significantly increased with to be male gender. In multivariate analysis, adjusting for age, marital status and type of blood donation, education level (OR, 1.17 [95%CI, 1.05–1.31], *p = 0.02*) and male gender (OR, 1.37 [95%CI, 1.14–1.65], *p = 0.005*) were associated with HBV-infection. HCV-prevalence was 2.32% and significantly increased with living outside Bamako. In multivariate analysis, adjusting for gender, age and education level, living outside Bamako was associated with HCV-infection (OR, 1.83 [95% CI, 1.41–2.35], *p < 0.001*). Syphilis seroprevalence was very low (0.04%) with only 3 individuals infected. Contrary to a prior study, blood donation type was not, after adjustments, an independent risk factor for each infection.

**Conclusions:**

Overall, HIV and HBV infection was higher in individuals with a lower level of education, HBV infection was higher in men, and HCV infection was higher in people living outside of Bamako. Compared to studies performed in 1999, 2002 and 2007 in the same population, we found that HIV and HCV prevalence have decreased in the last two decades whereas HBV prevalence has remained stable. Our finding will help guide infection prevention and treatment programs in Mali.

## Background

Human Immunodeficiency virus (HIV), Hepatitis B virus (HBV) and Hepatitis C virus (HCV) infections remain a global public health concern especially in Africa [1, 2]. West and central Africa region accounts for 21% of the world’s new HIV-infections and 30% of global deaths from AIDS-related illness [3]. Both the rate of new HIV-infections and the burden of HIV remain high in western and central Africa. The incidence prevalence ratio in the region has changed little since 2010, it stood at 0.06 [0.04–0.09] in 2017, twice as high as the epidemic transition benchmark of 0.03 [3]. Deaths from AIDS-related illness in the region have declined by nearly a quarter since 2010, and annual new HIV-infections declined by 8%. Cameroon, Ivory Coast and Nigeria together accounted for approximately 71% of new HIV-infections in the region in 2017 [3]. The World Health Organization (WHO) estimated that globally in 2015, viral hepatitis led to 1.34 million deaths and 96% were the results of complication of chronic HBV (66%) and HCV (33%) infections [4]. In African region, prevalence of this two virus infections was respectively 6.1% (about 60 million people) and 1% (about 11 million people) [4]. In 2019, Hepatitis Scorecard 2019 for WHO Africa Region reported HBV and HCV prevalence for various countries located in West Africa: Nigeria 5.5 and 2.1%, Ivory Coast 6.1 and 1.7%, Cameroon 4.4 and 0.7% or Mali 8.5 and 3.1% respectively [5]. Otherwise, Syphilis prevalence in Africa varies according to population study; among blood donors in Nigeria prevalence was 3.1% [6] whereas in a population of men who have sex with men (MSM) living in Burkina Faso, 6.1% were tested positive for *Treponema pallidum* antibodies [7].

Previous studies including blood donors from Bamako reported that HIV and HBV prevalence were respectively 4.5 and 14.9% in 2002 and 2.6 and 13.9% in 2007 [8, 9], HCV prevalence was 4% in 1998/1999 and 3.3% in 2007 [9, 10] and Syphilis infection prevalence was 0.3% [9]. This cross-sectional study aimed to estimate recently HIV, HBV, HCV and syphilis infections from blood donors in Bamako, Mali and to identify risk factors associated with each infection because no data are available yet.

## Methods

### Study population

We conducted a cross-sectional study of individuals donating blood from January 1st 2018 and December 31st 2018 to the blood bank of Gabriel Toure University teaching hospital in Bamako, Mali. We aimed to estimate HIV, HBV, HCV and syphilis infection prevalence and risk factors of blood donors during this time. The current study included all blood donations during 2018 (*n* = 8207) and respecting blood donation criteria: age between 18 and 60 years, body weight above 55 kg and physically healthy. Excluded from donation were individuals with known chronic diseases, high blood pressure, anemia, not transfused in the last 10 years, not hospitalized in the last 3 months, and not addicted to intravenous or oral drugs.

For each blood donor, socio-demographic data including age, marital status, education level, geographical setting and type of blood donation (volunteer or parental) were collected during a predonation examination.

### HIV, HBV, HCV and syphilis testing

To assure the safety of blood products transfused to patients at Gabriel Toure teaching hospital, all donations are analyzed for HIV-1/HIV-2 antibodies, HCV antibodies, hepatitis B surface antigen (AgHBs) and *T. pallidum* (TPHA/VDRL). Testing was performed on blood tubes taken after each donation, and consisted of the measurement of antibodies for HIV-1 and HIV-2 (Genscreen VIH1/2 version 2, BioRad, France), HCV (Murex, Abbott, France), hepatitis B surface antigen (HBsAg) (Monolisa AgHBS plus, BioRad, France) and *T. pallidum* (VDRL). All initial positive results were confirmed by performing of a second test using the same assay on a blood sample from a second phlebotomy. Confirmatory or supplemental testing, such as western blot for HIV, was not performed since such testing is not practice in the blood donation algorithm in Mali. Thus, data for these analyses were obtained from individual blood donors who had infection testing performed as part of routine practice to screen the blood supply at this hospital in Mali.

### Statistical analysis

Continuous variables were described with median and interquartile range [IQR] and categorical variables as number and percentages. The prevalence of HIV, HBV and HCV was expressed with a 95% confidence interval (CI95%) and group’s comparison was performed using Chi-2 test for categorical variables. Univariable and multivariable (including gender, age, education level (none, primary school, secondary school, higher), marital status, geographical setting and type of blood donation covariates) logistic regression analyses were performed with R (v3.6.1) [11] software to identify risk factors of each infection. Factors associated with HIV, HBV or HCV infection with a *P* value of < 0.20 in the univariate logistic regression analyses were included in the multiple logistic regression model. The level of significance for each analysis was set at 0.05.

## Results

### Socio-demographic data

From 8207 blood donors, 148 were excluded due to missing HIV, HBV and HCV status; final analysis included 8059 blood donors with a median age of 30 [25–38] years, 7157 (88.8%) men, 5500 (68.2%) married, 6096 (75.6%) living in Bamako, 4749 (58.9%) reached primary school or less, and 7898 (98.0%) parental donors.

### HIV, HBV and HCV prevalence

HIV-seroprevalence was 2.16% (95% CI: 1.86–2.50) with no significant difference between men (2.15%) and women (2.22%) (*p = 0.90*). HIV-prevalence increased with age (*p = 0.06*), from 1.86% in the youngest category (18–30 years old) to 3.40% in the oldest (> 50 years old). HIV-prevalence also varied by marital status, with a significantly higher prevalence for those who were married (2.42%) compared to single (1.54%) (*p = 0.013*). People living in Bamako also tended to have lower HIV-prevalence than those living in surrounding areas (2.00% versus 2.66%, respectively) (*p* = *0.08*). Otherwise, higher HIV-seroprevalence was observed with decreasing education level (*p = 0.0024*). None of the voluntary donors were HIV-infected (*p = 0.05*) (Table 1).

**Table 1:** HIV, HBV and HCV prevalence, according to sociodemographic and geographical data, from Bamako blood bank, year 2018

Characteristics	Total	HIV +		*Pvalue*	HBV +		*Pvalue*	HCV +		*Pvalue*
		No	% (CI 95%)		No	% (CI 95%)		No	% (CI 95%)	
All	8059	174	2.16 (1.86–2.50)	*–*	1191	14.78 (14.02–15.57)	*–*	187	2.32 (2.01–2.67)	*–*
Men	7157	154	2.15 (1.84–2.51)	*0.90*	1084	15.15 (14.33–16.0)	*0.0088*	172	2.40 (2.07–2.78)	*0.16*
Women	902	20	2.22 (1.44–3.40)		107	11.86 (9.91–14.14)		15	1.66 (1.01–2.73)	
Age group (years)										
18–30	4088	76	1.86 (1.49–2.32)	*0.06*	623	15.24 (14.17–16.37)	*0.10*	99	2.42 (1.99–2.94)	*0.005*
31–40	2525	62	2.46 (1.92–3.14)		371	14.69 (13.37–16.13)		54	2.14 (1.64–2.78)	
41–50	1168	27	2.31 (1.59–3.34)		157	13.44 (11.61–15.52)		20	1.71 (1.11–2.63)	
> 50	265	9	3.40 (1.80–6.33)		35	13.21 (9.65–17.81)		14	5.28 (3.17–8.67)	
Education level										
None	8	1	12.50 (0.64–47.09)	*0.0046*	0	0.00 (0.00–32.44)	*0.15*	0	0.00 (0.00–32.44)	*0.10*
Primary	4741	121	2.55 (2.14–3.04)		728	15.36 (14.36–16.41)		124	2.62 (2.20–3.11)	
Secondary	1416	24	1.70 (1.14–2.51)		197	13.91 (12.21–15.81)		25	1.77 (1.20–2.59)	
Higher	1724	25	1.45 (0.98–2.13)		242	14.03 (12.48–15.76)		35	2.03 (1.46–2.81)	
Marital status										
Single	2466	38	1.54 (1.13–2.11)	*0.013*	390	15.81 (14.43–17.31)	*0.08*	56	2.27 (1.75–2.94)	*0.80*
Married	5500	133	2.42 (2.04–2.86)		787	14.31 (13.41–15.26)		130	2.36 (1.99–2.80)	
Geographical setting										
Bamako	6096	122	2.00 (1.68–2.38)	*0.08*	904	14.83 (13.96–15.74)	*0.82*	116	1.90 (1.58–2.28)	*< 0.0001*
Around Bamako	1955	52	2.66 (2.03–3.47)		286	14.63 (13.13–16.27)		71	3.63 (2.89–4.55)	
Type of blood donation										
Parental donors	7898	174	2.20 (1.90–2.55)	*0.05*	1160	14.69 (13.92–15.49)	*0.15*	184	2.33 (2.02–2.69)	*> 0.99*
Volunteers donors	160	0	0.00 (0.00–2.35)		30	18.75 (13.46–25.06)		3	1.88 (0.51–5.37)	

CI: confidence interval; HBV: Hepatitis B virus; HCV: Hepatitis C virus; HIV: Human Immunodeficiency virus; No: number

HBV-prevalence was 14.78% (95% CI: 14.02–15.57) and was significantly higher in men (15.15%) than in women (11.86%) (*p = 0.0088*). We found no significant differences in HBV-prevalence by age, marital status, geographical setting, education level or type of blood donations (Table 1).

HCV-prevalence was 2.32% (95% CI: 2.01–2.67) with no significant differences between men (2.40%) and women (1.66%) (*p = 0.16*). HCV-prevalence was significantly different by age (*p = 0.005*) with the highest HCV prevalence observed among individuals older than 50 years (5.28%). Comparing geographical setting, HCV-prevalence was statistically higher for those living around Bamako (3.63%) than in Bamako (1.90%) (*p < 0.0001*). Marital status, education level and type of blood donations were not associated with HCV-prevalence variation (Table 1).

### Co-infection prevalence

In our study, 33 (0.41, 95% CI: 0.29–0.57)), 7 (0.09, 95% CI: 0.042–0.18) and 32 (0.40, 95% CI: 0.28–0.56) blood donors were co-infected with HIV/HBV, HIV/HCV and HBV/HCV, respectively. Among HIV/HBV, HIV/HCV and HBV/HCV groups, 90.91% (30), 85.71% (6) and 93.75% (30) were men, respectively. Except for HIV/HCV co-infection, the majority of co-infected blood donors were 18 to 30 years old (HIV/HBV: 48.48% (16) and HBV/HCV: 62.50% (20)). The majority of co-infected blood donors reached primary school or less, were married and lived in Bamako (HIV/HBV, respectively 72.73% (24), 78.79% (26) and 63.64% (21); HIV/HCV, respectively 71.43% (5), 85.71% (6) and 42.86% (3); HBV/HCV, respectively 68.75% (22), 65.63% (21) and 59.38% (19)). Of note, all co-infected patients were parental donors (Table 2).

**Table 2:** HIV, HBV and HCV co-infection prevalence, according to sociodemographic and geographical data, from Bamako blood bank, year 2018

CHARACTERISTICS	Total	HIV/HVB co-infection		HIV/HCV co-infection		HBV/HCV co-infection	
		No	% (CI 95%)	No	% (CI 95%)	No	% (CI 95%)
All	8059	33	0.41 (0.29–0.57)	7	0.09 (0.042–0.18)	32	0.40 (0.28–0.56)
Men	7157	30	0.42 (0.29–0.60)	6	0.08 (0.038–0.18)	30	0.42 (0.29–0.60)
Women	902	3	0.33 (0.09–0.98)	1	0.11 (0.006–0.62)	2	0.22 (0.039–0.81)
Age group (years)							
18–30	4088	16	0.39 (0.24–0.64)	1	0.02 (0.001–0.14)	20	0.49 (0.32–0.76)
31–40	2528	12	0.48 (0.27–0.83)	3	0.12 (0.032–0.35)	11	0.44 (0.24–0.78)
41–50	1168	4	0.34 (0.13–0.88)	2	0.17 (0.03–0.62)	1	0.09 (0.004–0.48)
> 50	265	1	0.38 (0.02–2.10)	1	0.38 (0.019–2.11)	0	0.00 (0.00–1.43)
Education level							
None	8	0	0.00 (0.00–32.44)	0	0 (0.00–32.44)	0	0.00 (0.00–32.44)
Primary	4741	24	0.51 (0.34–0.75)	5	0.11 (0.045–0.25)	22	0.46 (0.31–0.70)
Secondary	1416	4	0.28 (0.11–0.72)	1	0.07 (0.004–0.40)	1	0.07 (0.004–0.40)
Higher	1724	5	0.29 (0.12–0.68)	1	0.06 (0.003–0.33)	8	0.46 (0.24–0.91)
Marital status							
Single	2466	7	0.28 (0.14–0.59)	1	0.04 (0.002–0.23)	11	0.45 (0.25–0.80)
Married	5500	26	0.47 (0.32–0.69)	6	0.11 (0.05–0.24)	21	0.38 (0.25–0.58)
Geographical setting							
Bamako	6096	21	0.34 (0.23–0.53)	3	0.05 (0.013–0.15)	19	0.31 (0.20–0.49)
Around Bamako	1955	12	0.61 (0.35–1.07)	4	0.21 (0.08–0.53)	13	0.67 (0.39–1.13)
Type of blood donation							
Parental donors	7898	33	0.42 (0.30–0.59)	7	0.09 (0.043–0.18)	32	0.41 (0.29–0.57)
Volunteers donors	160	0	0.00 (0.00–2.35)	0	0.00 (0.00–2.35)	0	0.00 (0.00–2.35)

CI: confidence interval; HBV: Hepatitis B virus; HCV: Hepatitis C virus; HIV: Human Immunodeficiency virus; No: number

### Syphilis prevalence

Only 3 (0.04%, [95% CI, 0.01–0.11]) blood donors tested positive for syphilis. All were men, younger than 30 years and parental donors. Two were single and lived in Bamako. One of them was co-infected with HIV.

### Risk factors associated with HIV, HBV and HCV infections

Univariate and multivariate logistic regression analyses were performed to assess independent associations between socio-demographic data and HIV, HBV or HCV infections. In univariate analysis, HIV was significantly associated with being married (odds ratio [OR], 1.57 [95%CI, 1.16–2.15], *p = 0.016*) and education level lower than secondary school (OR, 1.69 [95% CI, 1.28–2.25], *p = 0.002*). HIV also tended to be associated with greater age (OR, 1.01 [95%CI, 1.00–1.03], *p = 0.06*). In multivariable analysis, HIV was only associated with education level (OR, 1.54 [95%CI, 1.15–2.07], *p = 0.016*). In univariate analysis, HBV-infection was significantly associated with being male (OR, 1.32 [95%CI, 1.10–1.59], *p = 0.013*) and this result was consistent in multivariable analysis (OR, 1.37 [95%CI, 1.14–1.66], *p = 0.005*) adjusting for age, education level, marital status and type of blood donation. Education level lower than secondary school was also associated with HBV in multivariable analysis (OR, 1.17 [95%CI, 1.05–1.31], *p = 0.021*). Living outside Bamako was the only factor associated with HCV in multivariable analysis adjusting for gender, age and education level (OR, 1.83 [95%CI, 1.41–2.35], *p = 0.0001*) (Table 3).

**Table 3:** Risks factors associated with HIV, HBV and HCV infection in blood donors’ population from Bamako, Mali, year 2018

RISK FACTORS	UNIVARIATE ANALYSIS			MULTIVARIATE ANALYSIS		
	OR	CI 95%	*P-value*	OR	CI 95%	*P-value*
HIV INFECTION						
Gender (reference group: women)	0.96	0.65–1.47	*0.86*	–	–	–
Age	1.01	1.00–1.03	*0.06*	1.00	0.99–1.02	0.71
Education level None and primary vs secondary and higher	1.69	1.28–2.25	0.002^**^	1.54	1.15–2.07	0.016^*^
Marital status (reference group: single)	1.57	1.16–2.15	0.016^*^	1.33	0.94–1.92	0.18
Geographical setting (reference group: Bamako)	1.31	0.99–1.73	*0.11*	1.23	0.92–1.62	0.24
Type of blood donation (reference group: volunteer)	3.5 10^5^	60–6.8 10^48^	*0.97*	–	–	–
HBV INFECTION						
Gender (reference group: women)	1.32	1.10–1.59	0.013^*^	1.37	1.14–1.66	0.005^**^
Age	0.99	0.99–1.0	*0.05*	0.99	0.99–1.00	0.13
Education level None and primary vs secondary and higher	1.11	1.00–1.24	*0.11*	1.17	1.05–1.31	0.021^*^
Marital status (reference group: single)	0.89	0.80–1.00	*0.085*	0.93	0.81–1.06	0.35
Geographical setting (reference group: Bamako)	0.97	0.86–1.10	*0.69*	–	–	–
Type of blood donation (reference group: volunteer)	0.61	0.38–1.01	*0.092*	0.57	0.36–0.96	0.06
HCV INFECTION						
Gender (reference group: women)	1.38	0.90–2.22	*0.24*	1.38	0.90–2.22	0.24
Age	1.01	1.00–1.02	*0.19*	1.00	0.99–1.02	0.52
Education level None and primary vs secondary and higher	1.38	1.07–1.80	0.043^*^	1.29	0.99–1.69	0.12
Marital status (reference group: single)	1.06	0.81–1.39	*0.73*	–	–	–
Geographical setting (reference group: Bamako)	1.90	1.47–2.44	< 0.0001^***^	1.83	1.41–2.35	0.0001^***^
Type of blood donation (reference group: volunteer)	1.61	0.43–15.41	*0.64*	–	–	–

CI: confidence interval; HBV: Hepatitis B virus; HCV: Hepatitis C virus; HIV: Human Immunodeficiency virus; No: number; −: parameter not included in multivariate analysis; * < 0.05; ** < 0.01; *** < 0.001

## Discussion

This cross-sectional study aimed to measure prevalence and risk factors for HIV, HBV, HCV, and syphilis infections from blood donors in Bamako, Mali.

HIV-seroprevalence was independently associated with a decreasing education level as has been observed in prior studies from several regions of the world [12]. Prior research suggests that knowledge about HIV/AIDS increases with both level and quality of education [12, 13], and consequently, higher education level has been associated with less HIV/AIDS risk taking behavior [14]. For example, a study reported that for every additional year in education, there was greater use of condoms [14]. Conversely, low education level was associated with higher likelihood of engaging in transactional sex (drugs or money), as well as neighbor’s education level, and thus increased HIV acquisition risk [15]. HBV-prevalence was higher in men and blood donors with educational level lower than secondary school. Similar results were reported in Burkina Faso where HBV-seroprevalence was significantly higher in men than in women (10.5% vs 7.8%) [16]. This finding could be due to better access to HBV vaccination for women accessing medical care during pregnancy. But it may also related to different T-cell immunity between gender; indeed, women were reported to exhibit greater humoral and cell-mediated responses to antigenic stimulation, vaccination and infection than do men [17]. We also found that HCV was more prevalent in blood donors living outside of Bamako; this could be related to unsafe non aseptic practices putting individuals in rural areas at risk for HCV such as female excision (removal of a part or entire clitoris), circumcision or cultural scarification as previously reported in others African countries [18] but probably not linked to injection drugs users mainly present in the center of Bamako. None of these specific factors were assessed in this study and further investigation should be undertaken to completely assess HCV epidemiology and transmission knowledge in Mali. Finally as expected, syphilis prevalence was very low with only 3 blood donors infected with *Treponema pallidum.* Although sexual orientation was not collected in our study, a recent study performed in Burkina Faso reported that the prevalence of syphilis among MSM was also low and comparable to that of other reproductive age individuals, suggesting that public health intervention programs were effective [7].

Although HIV-prevalence was significantly higher in parental donors compared to volunteer donors (2.20 vs 0.00, *p = 0.05*), HBV and HCV prevalence were not different between the parental and voluntary donors (14.69 vs 18.75, *p = 0.15* and 2.33 vs 1.88, *p > 0.99*, respectively) and in multivariate analysis, parental donor was not associated with any of these infections. A prior study performed in the National Centre for Blood Transfusion (CNTS) of Mali located in Bamako reported higher prevalence of HIV and HBV in parental blood donors suggesting that number of regular, volunteer blood donors should be increased [9]. However, they didn’t perform multivariate logistic regression analysis and thus blood donation type should not be considered as an independent risk factor for each infection.

**Fig. 1:**
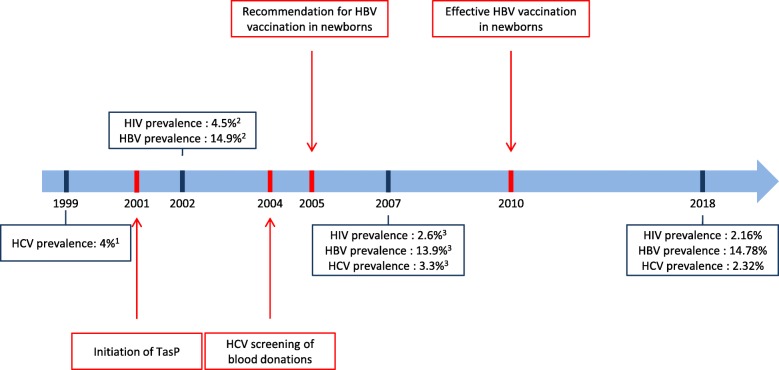
HIV, HBV and HCV prevalence during the last two decades in Bamako blood bank and medical improvements implemented*.* In blue are represented all information’s about HIV, HBV and HCV prevalence in 1999, 2002, 2007 and 2018 and in red, medical interventions that could explain reduction of HIV and HCV-prevalence in blood donors. TasP: treatment as prevention, ^1^Dembele et al., Bull Soc Pathol Exot, 2004; ^2^Tounkara et al., J Int Assoc Physicians AIDS Care, 2009; ^3^Diarra et al., Transfus Clin Biol, 2009.

HIV, HBV and HCV prevalence were previously evaluated in a blood donor population from CNTS of Bamako in 1999, 2002 and 2007 [8–10] (Fig. 1). Comparing across time periods, HIV-prevalence has decreased from 4.5% in 2002 to 2.6% in 2007, and 2.16% in 2018. This reduction in HIV-prevalence is likely multi-factorial, but could be influenced by the initiation of the large scale antiretroviral treatment programs since 2001 in Mali. In contrast, HBV-prevalence was stable at 14.9% in 2002, 13.9% in 2007, and 14.78% in 2018. This is despite recommendations in 2005 for HBV vaccination in all newborns [19], as this program was not fully initiated in Mali until 2010. Thus, the benefit of widespread childhood HBV vaccination is not yet evident. As with HIV-prevalence, HCV prevalence also decreased in the last two decades, from 4% in 1999, to 3.3% in 2007 and 2.28% in 2018. This decrease can likely be attributed to the systematic screening of all blood donors for HCV since 2004 but also by the initiation of HIV-infection prevention and treatment program since 2001, whose transmission route is similar to HCV-infection.

## Conclusion

Although benefit of HBV vaccination is not yet evident, HIV and HCV prevalence have decreased during the last two decades. None of this prevalence was related to the type of blood donation. Our finding may help officials and groups planning infection and care programs in Mali.

## Data Availability

The data presented in this study were collected from the data bank of blood donors available at Gabriel Toure University teaching hospital in Bamako, Mali.

## Abbreviations

AIDS: Acquired Immunodeficiency Syndrome
CI95%: 95% Confidence Interval
CNTS: National Centre for Blood Transfusion
HBV: Hepatitis B virus
HCV: Hepatitis C virus
HIV: Human Immunodeficiency virus
IQR: Interquartile Range
MSM: Men who have Sex with Men
OR: Odds Ratio
TPHA: *Treponema pallidum* Haemagglutination Assay
VDRL: Veneral Disease Reagent Laboratory
WHO: World Health Organization
